# Neutrophils as Suppressors of T Cell Proliferation: Does Age Matter?

**DOI:** 10.3389/fimmu.2019.02144

**Published:** 2019-09-11

**Authors:** Cathelijn E. M. Aarts, Ida H. Hiemstra, Anton T. J. Tool, T. K. van den Berg, Erik Mul, Robin van Bruggen, Taco W. Kuijpers

**Affiliations:** ^1^Department of Blood Cell Research, Sanquin Research, Amsterdam University Medical Center, University of Amsterdam, Amsterdam, Netherlands; ^2^Department of Research Facilities, Sanquin Research Amsterdam, Amsterdam, Netherlands; ^3^Department of Pediatric Immunology, Rheumatology and Infectious Diseases, Emma Children's Hospital, AUMC, University of Amsterdam, Amsterdam, Netherlands

**Keywords:** neutrophils, progenitors, MDSC activity, T cell suppression, cell isolation

## Abstract

Whereas, neutrophils have long been considered to mainly function as efficient innate immunity killers of micro-organisms at infected sites, they are now recognized to also be involved in modulation of adaptive immune responses. Immature and mature neutrophils were reported to have the capacity to suppress T cell-mediated immune responses as so-called granulocyte-myeloid-derived suppressor cells (g-MDSCs), and thereby affect the clinical outcome of cancer patients and impact the chronicity of microbial infections or rejection reactions in organ transplantation settings. These MDSCs were at first considered to be immature myeloid cells that left the bone marrow due to disease-specific signals. Current studies show that also mature neutrophils can exert suppressive activity. In this study we investigated in a robust T cell suppression assay whether immature CD11b+ myeloid cells were capable of MDSC activity comparable to mature fully differentiated neutrophils. We compared circulating neutrophils with myeloid cell fractions from the bone marrow at different differentiation stages. Our results indicate that functional MDSC activity is only becoming detectable at the final stage of differentiation, depending on the procedure of cell isolation. The MDSC activity obtained during neutrophil maturation correlated with the induction of the well-known highly mobile and toxic effector functions of the circulating neutrophil. Although immature neutrophils have been suggested to be increased in the circulation of cancer patients, we show here that immature neutrophils are not efficient in suppressing T cells. This suggests that the presence of immature neutrophils in the bloodstream of cancer patients represent a mere association or may function as a source of mature neutrophils in the tumor environment but not a direct cause of enhanced MDSC activity in cancer.

## Introduction

Almost 20 years ago the expansion of immature myeloid cells in bone marrow, spleen and the circulation was described in cancer patients and many animal models (1–6). These cells were found to have a suppressive effect on T cell proliferation and were therefore named myeloid-derived suppressor cells (MDSCs) (7). It is thought that under pathological conditions, such as cancer, a partial block occurs in the differentiation of immature myeloid cells, resulting in the expansion of this population. Their presence and infiltration have been associated with poor prognosis (8–10). There are two subsets of MDSCs described, namely the monocytic-MDSCs (M-MDSC) and the granulocytic or polymorphonucelar-MDSCs (G- or PMN-MDSCs), which are developmentally immature and found at different stages of myelopoiesis (11–13). The g-MDSC were found to be closely related to neutrophils and are predominantly defined by their capacity to suppress T cell activation and proliferation (3, 5, 14). In fact, many studies have found that also mature neutrophils are able, under certain conditions, to suppress the T cells proliferation. The view of g-MDSCs by some is now more considered as a phenotype or subset of neutrophils (15, 16). Therefore, we wondered if the developmental stage is crucial for neutrophils to exert MDSC activity (i.e., T cell suppression), or in other words, whether “age” of a neutrophil would matter.

In a recent study, we investigated in more depth the mechanism of MDSC activity of mature neutrophils of healthy donors. Here we have shown that MDSC activity is only present upon activation and is dependent on ROS production and release of granule components in a CD11b-dependent manner, facilitated by trogocytosis (Aarts et al. under review). Using the same model, we investigated the capacity of human immature neutrophils derived from the bone marrow to exert MDSC activity. We isolated the different neutrophil progenitors from bone marrow, defined by their expression of the surface markers CD11b and CD16. Isolation of cells based on FACS sorting is common practice, also in the field of MDSC research. Many studies rely on this method to isolate MDSCs. However, we found that FACS sorting caused a major impairment on the functionality of the cells, where also mature neutrophils from blood were not able to perform classical functions upon activation by physiological stimuli such as chemoattractants. Therefore, to circumvent the use of FACS sorting, we isolated the progenitors by density centrifugation, followed by CD16-positive magnetic-activated cell sorting (MACS). We found that only the most mature progenitor neutrophils were able to suppress the T cell proliferation, suggesting that MDSCs are perhaps indeed more a phenotype of neutrophils.

## Materials and Methods

### Study Approval

Bone marrow samples were obtained from individuals considered to be healthy, undergoing surgery for unrelated, non-hematological procedures after obtaining written informed consent in accordance with the Declaration of Helsinki (version Seoul 2008). Heparinized peripheral blood samples were collected from healthy donor volunteers.

### Cell Isolation

Bone marrow or peripheral blood was diluted 1:1 with PBS/TSC (tri-sodium citrate) and separated based on density by centrifugation over isotonic Percoll (Pharmacia, Uppsala, Sweden) with a specific density of 1.076 g/mL. The interphase fraction derived from the peripheral blood, containing peripheral blood mononuclear cells (PBMC), was harvested for isolation of untouched T cells by magnetic-activated cell sorting with the Pan T cell isolation kit of Miltenyi-Biotec (Bergisch Gladbach, Germany) according to the manufacturer's instructions. The pellet fractions from total bone marrow or whole blood samples contained the neutrophil progenitors or mature neutrophils, respectively. The pellet fractions and total bone marrow samples underwent erythrocyte lysis with hypotonic ammonium chloride solution at 4°C. The cells were kept in Hepes-buffered saline solution (HBSS containing 132 mM NaCl, 6 mM KCl, 1 mM CaCl_2_, 1 mM MgSO_4_, 1.2 mM potassium phosphate, 20 mM Hepes, 5.5 mM glucose, and 0.5% (w/v) human serum albumin, pH 7.4).

Neutrophil progenitors from total bone marrow were separated by FACS sorting based on FSC/SSC and the expression of CD11b (APC-labeled, clone D12, BD Biosciences) and CD16 (PE-labeled, clone 3G8, BD Biosciences). Neutrophils isolated from whole blood were sorted either based on only FSC/SSC or also in combination with the expression of CD11b and CD16. FACS sorting was performed with either a small (85 μM) or big (100 μM) nozzle under cold or room temperature conditions using BD FACS Aria III (BD biosciences).

Neutrophil progenitors from bone marrow were isolated using discontinuous Percoll fractionation, as described previously (17). In short, after erythrocyte lysis bone marrow aspirate was placed upon a two-layer Percoll gradient of densities 1.065 and 1.080 g/mL and centrifuged at 2,000 rpm, acceleration 3, brake 0, for 20 min. After centrifugation the four cell fractions were collected, washed and resuspended in HEPES+ buffer.

CD16 positive cells were isolated from the pellet fractions after density centrifugation of whole blood or bone marrow by magnetic-activated cell sorting with human CD16 microbeads of Miltenyi-Biotec according to the manufacturer's instructions. The CD16 negative cells were collected from the flow through.

### Antibodies and Flow Cytometry

The following directly conjugated antibodies were used for flow cytometry analysis: PB-labeled anti-CD11b (clone ICRF44, BD Biosciences), PECy7-labeled anti-CD16 (clone 3G8, BD Biosciences), FITC-labeled anti-FPR1 (clone 350418, R&D systems), FITC-labeled anti-fMLP, PE-labeled anti-TLR4 (clone 610015, R&D systems), PE-labeled TNFR1 (clone 16803, R&D systems), APC-labeled anti-TNFR2 (clone 22235, R&D systems).

Flow cytometry data were acquired using Canto II flow cytometer (BD Biosciences) and analyzed using FlowJo software (Tree Star, USA).

### T Cell Proliferation Assay

Purified T cells were labeled with CFSE (Molecular probes, Life Technologies, Carlsbad, CA, USA) and cultured in 96-well flat bottom plates (Nunclon Delta Surface, Thermo Scientific, Waltham, MA, USA) for 5 days—or otherwise if indicated—at 37°C in IMDM medium (Gibco, Life Technologies, Carlsbad, CA, USA), supplemented with 10% (v/v) fetal calf serum (Bodinco, Alkmaar, The Netherlands), 10^4^ U/mL penicillin, 10 ng/mL streptomycin, 200 mM glutamine, and 0.035% (v/v) β-mercaptoethanol (Sigma-Aldrich, Saint Louis, MO, USA). Proliferation was induced by anti-CD3 (clone 1XE [IgE isotype] hybridoma supernatant, 1:1,000, Sanquin, Amsterdam, The Netherlands) and anti-CD28 (clone 15E8 [IgG1 isotype] at 5 μg/mL, Sanquin) monoclonal antibodies (moAbs; at 20,000 T cells/well). Mature neutrophils from blood or neutrophil progenitors from bone marrow were added in a 1:3 ratio (60,000 neutrophils/well), in the presence or absence of neutrophil-activating stimuli: fMLF (1 μM, Sigma-Aldrich), TNFα (10 ng/mL, Peprotech EC, London, UK) or LPS (20 ng/mL, *E. coli* 055:B5, Sigma).

After 4–6 days, T cell proliferation, indicated by CFSE dilution, was analyzed by flow cytometry. Cells were harvested from the culture plates and stained with APC-labeled anti-CD4 (clone SK3, BD Biosciences, San Jose, CA, USA) and PerCPCy5.5-labeled anti-CD8 (clone SK1, Biolegend, San Diego, CA, USA) antibodies.

### ROS Production

NADPH oxidase activity was measured by assaying the hydrogen peroxide production by mature neutrophils derived from blood or neutrophil progenitors derived from bone marrow in response to various stimuli with the Amplex Red kit (Molecular Probes, Life Technologies, Carlsbad, CA, USA). Cells (1 × 10^6^/mL). In short, cells were stimulated in Hepes-buffered saline solution with fMLF (1 μM), TNFα (10 ng/mL), LPS (20 ng/mL) + LPS-binding protein (LBP) (50 ng/mL, R&D Systems, Minneapolis, MN, USA) or PMA (100 ng/mL, Sigma) in the presence of Amplex Red (0.5 μM) and horseradish peroxidase (1 U/mL). Fluorescence was measured at 30 s intervals during 4 h with the HTS7000+ plate reader (Tecan, Zurich, Switzerland). Maximal slope of hydrogen peroxide release was assessed over a 2 min interval.

### Cytospins and Staining

0.5 or 1 × 10^5^ cells were cytospun (Shandon CytoSpin II Cytocentrifuge) onto 76 × 26 mm glass microscope slides. The slides were air-dried and stained. Cytospins slides were incubated for 5 min in May-Grünwald followed by 15 min in phosphate buffer and subsequent Giemsa solution staining for 30 min. Slides were rinsed in deionized water, air-dried and analyzed with Zeiss Scope.A1 microscope.

### Statistics

Statistical analysis was performed with GraphPad Prism version 7 for Windows (GraphPad Software, San Diego, CA, USA). Data were evaluated by one-way ANOVA or unpaired two-tailed student's *t*-test. The results are presented as the mean ± SEM. Data were considered significant when *p* < 0.05.

## Results

### Sorted Isolated Neutrophil Progenitor Cells From Bone Marrow Do Not Exert MDSC Activity

**Figure 1:**
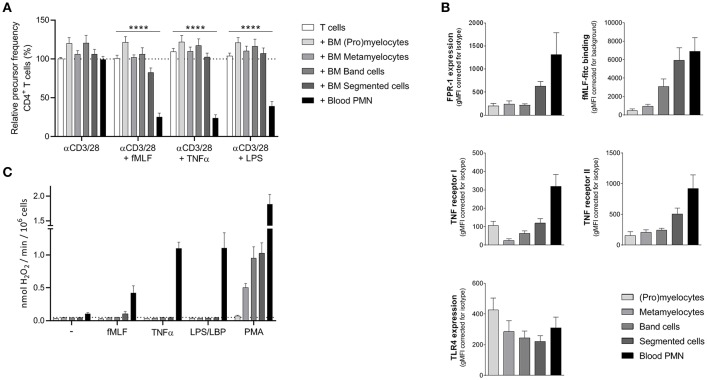
Sorted neutrophil progenitor cells from bone marrow do not exert MDSC activity. Neutrophil progenitors from bone marrow were isolated via FACS sorting based on CD11b and CD16 expression under cold conditions and with a small nozzle. **(A)** Purified CFSE-labeled T cells from healthy donors (*n* = 6) were cultured with anti-CD3 and anti-CD28 antibodies (white bars), and in presence of mature neutrophils from control donors (black bars, *n* = 6) or sorted neutrophil progenitors from bone marrow (gray bars, *n* = 3) and/or indicated stimuli. Cells were harvested after 5–6 days and analyzed by flow cytometry for CFSE dilution among CD4^+^ T cells. **(B)** The surface marker expression of FPR-1 and fMLF binding (top panel, *n* = 3–8), TNF receptor I and II (center panel, *n* = 4) and TLR4 (bottom panel, *n* = 4) was measured by flow cytometry analysis of mature neutrophils from blood (black bar) and neutrophil progenitors from bone marrow. **(C)** Mature neutrophils and neutrophil progenitors were stimulated with the indicated stimuli and production of H_2_O_2_ was determined by measuring Amplex Red conversion into fluorescent Resorufin (*n* = 3). Error bars indicate SEM; ^****^p < 0.0001.

As previously described (18), mature neutrophils from peripheral blood from healthy donors show MDSC activity (i.e., suppression of T cell proliferation), but only upon activation. To investigate whether immature neutrophils can also exert MDSC activity we tested neutrophil progenitor cells collected from bone marrow samples obtained from patients who underwent cardiac-bypass surgery unrelated to any cancer or underlying hematological or chronic inflammatory disease (other than atherosclerosis). Neutrophil progenitor cells can be divided in four different developmental stages, namely (pro)myelocytes, metamyelocytes, band cells, and segmented neutrophils (19). We isolated the progenitor cells based on the expression of surface markers CD11b and CD16 (Supplement Figure 1) via FACS sorting, which is also used as a method to isolate PMN-MDSC from the ring fraction after density gradient centrifugation (20). The four isolated neutrophil progenitor cells were cultured for 5 days in presence of isolated CFSE-labeled T cells from a healthy donor and were left unstimulated or activated with either fMLF, TNFα, or LPS. T cell proliferation was induced by monoclonal anti-CD3 and anti-CD28 antibodies and quantified as relative “precursor frequency”: i.e., percentage of naïve cells in the initial population that underwent one or more divisions upon anti-CD3/anti-CD28 antibodies (21), normalized for the condition with only stimulated T cells. See Supplement Figure 2 for the gating strategy of the flow cytometry analysis. As a control, mature neutrophils were isolated from the same healthy donor as the T cells. None of the isolated neutrophil progenitor cell fractions were able to suppress the T cell proliferation of CD4^+^ or CD8^+^ T cells (Figure 1A, Supplement Figure 3). Only the mature neutrophils isolated from blood were able to suppress the T cell proliferation upon activation. We hypothesized that maybe the lack of MDSC activity could be caused by an absent or lower expression of the receptors (FPR-1, TNF receptor, or TLR4). The expression of the fMLF and TNFα receptor is indeed increasing upon maturation in the bone marrow. However, the presence of the TLR4 is already present at the early progenitor stage onwards and is comparable to the expression of mature neutrophils isolated from blood (Figure 1B), indicating that the lack of MDSC activity upon LPS stimulation is not caused by the absence of the receptor as such.

One of the main effector mechanism of human MDSC activity that has been described in the literature is the generation of a large amount of reactive oxygen species (ROS) upon cell activation (Aarts et al. under review) (22). The sorted neutrophil progenitor cells were not able to produce ROS when activated by either fMLF, TNFα or LPS, which then may explain why these immature cells cannot suppress the T cell proliferation in our MDSC activity assay (Figure 1C).

### FACS Sorting Causes an Impairment in Neutrophil Function

**Figure 2:**
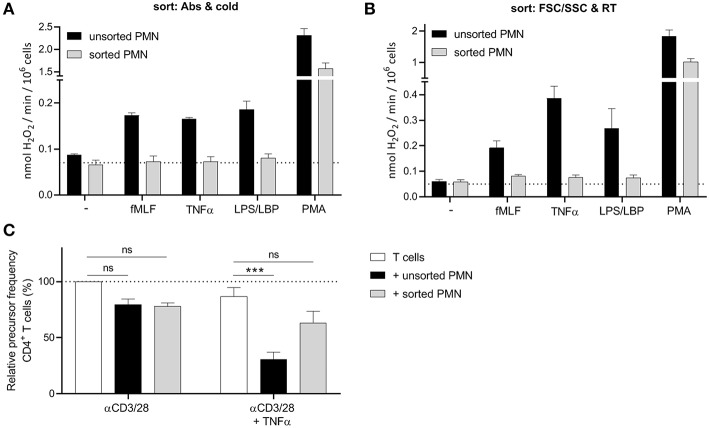
FACS sorting causes an impairment in neutrophil function. **(A,B)** Neutrophils were left unsorted (black bars, *n* = 6) or sorted based on CD11b and CD16 expression under cold conditions and a small nozzle (**A**, gray bars, *n* = 3) or based on size (FSC/SSC) under RT conditions and a big nozzle (**B**, gray bars, *n* = 5). Cells were stimulated with the indicated stimuli and production of H_2_O_2_ was determined by measuring Amplex Red conversion into fluorescent Resorufin. **(C)** Purified CFSE-labeled T cells from healthy donors were cultured with anti-CD3 and anti-CD28 antibodies (white bars), and in presence of unsorted (black bars) or sorted (gray bars) mature neutrophils from control donors and/or indicated stimuli (*n* = 3). Sort was based on size (FSC/SSC) under RT conditions and a big nozzle. Cells were harvested after 5–6 days and analyzed by flow cytometry for CFSE dilution among CD4^+^ T cells. Error bars indicate SEM; ***p < 0.001.

However, and much to our surprise, also the myeloid cells at the most mature progenitor stage in the bone marrow fractions (i.e., the segmented neutrophils of the “reserve pool”) were not able to produce ROS. This led us to believe that perhaps the sorting process to isolate the different progenitor cells may cause an impairment in cellular functions. Therefore, we tested multiple sorting conditions using mature neutrophils isolated from blood and investigated their ROS production after sorting. When we sorted mature neutrophils under the same conditions as our bone marrow samples (cold, antibody incubation at 4 degrees, Supplement Figure 1), we observed that the sorted neutrophil fraction was not able to produce ROS upon stimulation, apart from the non-physiological stimulus PMA, a phorbol ester that bypasses surface receptors and directly activates intracellular protein kinase C (Figure 2A).

We hypothesized, that the lack of ROS production may be caused by the pressure the cells undergo during sorting, since the temperature nor the labeling did impair the ROS production (Supplement Figure 4). To reduce the pressure during sorting we used the biggest nozzle (100 μM) and sorted the neutrophils only based on their size (FSC/SSC) and at room temperature to further exclude any other variable. Once again, the sorted neutrophils showed a lower ROS production and in addition also much less if any MDSC activity in our T cell proliferation assay (Figures 2B,C, Supplement Figure 5), concluding that FACS sorting is not an ideal method to isolate neutrophils and their progenitors from bone marrow for functional studies, including the detection of MDSC activity.

### Neutrophil Progenitors Isolated by Density Centrifugation Show a Less Effective MDSC Activity

**Figure 3:**
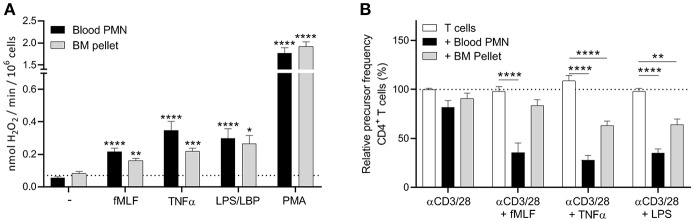
Neutrophil progenitors isolated by density centrifugation show a less effective MDSC activity. **(A)** Mature neutrophils (black bars) and neutrophil progenitors from the bone marrow pellet fraction after density centrifugation (gray bars) were stimulated with the indicated stimuli and production of H_2_O_2_ was determined by measuring Amplex Red conversion into fluorescent Resorufin (*n* = 3). **(B)** Purified CFSE-labeled T cells from healthy donors were cultured with anti-CD3 and anti-CD28 antibodies (white bars, *n* = 6), and in presence of mature neutrophils from blood (black bars, *n* = 6) or neutrophil progenitors from the bone marrow pellet (gray bars, *n* = 3) and/or indicated stimuli. Cells were harvested after 5–6 days and analyzed by flow cytometry for CFSE dilution among CD4^+^ T cells. Error bars indicate SEM; *p < 0.05, **p < 0.01, ***p < 0.001, ****p < 0.0001.

Since FACS sorting clearly had an impact on the functionality of neutrophils, we decided to isolate the progenitor cells by Percoll density centrifugation. The pellet fraction includes all the four progenitor populations, where the segmented neutrophils are the most abundant (around 50%, Supplement Figure 6). In contrast to the sorted progenitor fractions, the cells from the bone marrow (BM) pellet fraction were able to produce ROS upon stimulation (Figure 3A), indicating again that the sorting process indeed impair the cells. Also, in our T cell proliferation assay we observed some inhibition of the T cell proliferation by the BM pellet cells, though this was only observed in the proliferation of CD4^+^ T cells and not in the CD8^+^ T cells, where the suppression was not significant (Figure 3B, Supplement Figure 7). So to conclude, the ROS production and the MDSC activity of the BM pellet cells were not as effective as the mature neutrophils isolated from blood. This could be explained by the fact that the BM pellet fraction is still a heterogeneous cell population of immature (myelocytes and metamyleocytes) and early mature neutrophils (i.e., cells with a nuclear band and segmented shape), where the immature progenitor cells have a lower FPR-1 or TNF receptor expression. Therefore, to fully investigate the MDSC activity of the more immature progenitor cells an extra isolation step is needed for further functional testing.

### Only the CD16^+^ Neutrophil Progenitors Show MDSC Activity

Another technique to isolate the different neutrophil progenitor cells from bone marrow that has been described is discontinuous Percoll fractionation, where the increasing density of the cells with maturity forms the basis of the separation (17). Bone marrow aspirate was placed upon a two-layer Percoll gradient of densities 1.065 and 1.080 g/mL, generating four BM cell fractions after centrifugation (Supplement Figure 8A), where in the first fraction the most immature cells should end up and the most mature cells in the last fraction. However, when we analyzed the four obtained BM cell fractions with the maturation markers CD11b and CD16, we found that each BM cell fraction showed some cell heterogeneity where in each fraction segmented cells were present (Supplement Figures 8B,C). Apart from fraction 1, all the BM cell fractions were able to produce ROS upon different stimuli, including fMLF and TNFα which is surprising since the more immature neutrophil progenitor cells do not express the receptors of these ligands as highly as the more mature progenitors (Figure 1B). The presence of the ROS production may therefore be due to the presence of the mature segmented cells in the fractions. Because of this cell heterogeneity, we concluded that this method is not suitable for us to investigate the MDSC activity of neutrophil progenitor cells and need a different approach to separate the early immature from the more mature neutrophils in bone marrow.

To divide the progenitors from the BM pellet fraction into immature and “early mature” neutrophils, we isolated the CD16-positive cells from the BM pellet fraction by magnetic-activated cell sorting (MACS) with human CD16 microbeads. The CD16-positive neutrophil fraction from the BM pellet was depleted from the immature progenitors, however the CD16-negative fraction still included some “early mature” progenitors though this percentage was significantly lower than in the total BM pellet fraction and did not include the segmented cells as was the problem with the discontinuous Percoll fractionation (Supplement Figure 9).

**Figure 4:**
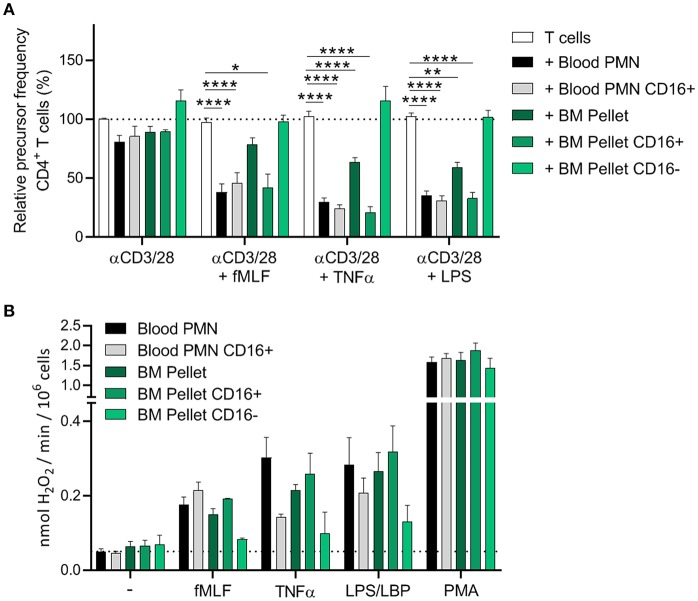
Only the CD16^+^ neutrophil progenitors show MDSC activity, ROS production and degranulation. **(A,B)** CD16 positive cells were isolated via MACS isolation from mature neutrophils from blood and neutrophil progenitors from BM pellet. **(A)** Purified CFSE-labeled T cells from healthy donors (*n* = 4) were cultured with anti-CD3 and anti-CD28 antibodies (white bars), and in presence of mature neutrophils from control donors (black bars, *n* = 6), CD16^+^ mature neutrophils (gray bars, *n* = 6), total BM pellet fraction (dark green bars, *n* = 4), CD16^+^ (green bars, *n* = 4) or CD16^−^ (light green bars, *n* = 4) progenitors from BM pellet and/or indicated stimuli. Cells were harvested after 5–6 days and analyzed by flow cytometry for CFSE dilution among CD4^+^ T cells. **(B)** The indicated cell fractions were stimulated with the indicated stimuli and production of H_2_O_2_ was determined by measuring Amplex Red conversion into fluorescent Resorufin (*n* = 4–6). Error bars indicate SEM; *p < 0.05, **p < 0.01, ****p < 0.0001.

The CD16-positive isolation procedure did not result in a less effective MDSC activity of mature neutrophils from blood (Figure 4A), indicating that MACS isolation is a more gentle approach to isolate cells than FACS sorting. Only the CD16-positive progenitor cells from the BM pellet were able to suppress the T cell proliferation (i.e., show MDSC activity). The CD16-negative progenitor cells showed an even higher T cell proliferation than T cells cultured without mature neutrophils or BM progenitor cells but definitely not any indication of enhanced MDSC activity (Figure 4A, Supplement Figure 10).

The CD16-positive and CD16-negative progenitor cells also differed in ROS production. The CD16-negative progenitors were hardly able to induce ROS production upon (physiological) stimulation, whereas the CD16-positive progenitors showed ROS production more comparable to circulating neutrophils isolated from blood. This could also explain why the total BM pellet shows a decent ROS production upon stimulation, since the BM pellet contains more of the “early mature” (band and segmented cells) neutrophils progenitors (Figure 4B).

Though the immature neutrophils seem to have the machinery to produce ROS, as was indicated by the PMA stimulation, the signal transduction pathways within these cells may not be fully developed yet to exert MDSC activity (23). When all these data are taken together, our results suggest that the MDSC activity obtained during neutrophil maturation in the bone marrow correlates with the induction of the well-known highly mobile and toxic effector functions of the fully differentiated neutrophil in the circulation.

## Discussion

MDSCs have been described as a heterogeneous subset of immature myeloid cells, defined by their capacity to suppress T cell activation and proliferation. It is thought that progenitor neutrophils exit the bone marrow early, migrate to blood and have suppressive activity as g-MDSCs with a role during the process of inflammation resolution (24). Tumors represent a chronic state of inflammation, and the presence and infiltration of MDSCs have been associated with poor prognosis (8–10). Although the numbers of immature neutrophils have been reported to be increased in the circulation of cancer patients, we show here that immature neutrophils *per se* are not efficient in suppressing T cells. Obviously, the neutrophil progenitors that we tested derive from patients who are not considered to be suffering from a chronic state of severe inflammation or cancer, which could explain their lack of MDSC activity. However, we were able to induce this activity in the “early mature” neutrophils from bone marrow fractions by activation with physiological neutrophil stimuli. In fact, we have shown in a recent study that MDSC activity by mature neutrophils from healthy donors can only be induced by certain and not all neutrophil activators (Aarts et al. under review), which correlates to the capacity to induce ROS production.

The expansion and accumulation of immature MDSCs in the bloodstream in presence of tumors have been often reported (1, 25). These immature neutrophils that are released in the circulation can perhaps function as a source for mature neutrophils, since it has been shown that immature neutrophils can mature in the circulation (26), or perhaps at the site of inflammation or within the tumor environment itself. It has been shown that adoptively transferred g-MDSCs can enter tumors and differentiate into mature phenotype (27). This could explain on the one hand a role for immature myeloid cells and at the same time explain why we could only observe MDSC activity in the more mature neutrophil and not in any of the immature progenitor fractions from bone marrow. Such a hypothesis is supported by our observation that MDSC activity obtained during neutrophil maturation correlated with the induction of motility and toxic effector functions of the circulating neutrophil required to suppress T cell proliferation (Aarts et al. under review) (22, 23, 28). We may suggest that under certain disease conditions, immature neutrophils can be released from the bone marrow where further differentiation takes place in the circulation or at the site of the inflammation or tumor development to become effective local T cell suppressor cells with MDSC activity.

## Data Availability

All datasets generated for this study are included in the manuscript/Supplementary Files.

## Ethics Statement

The studies involving human participants were reviewed and approved by Medische Ethische Toetsingcommissie, Amsterdam Universitair Medisch Centra (AUMC). The patients/participants provided their written informed consent to participate in this study.

## Author Contributions

TK and IH are the principle investigators, who conceived, and designed the study. CA, IH, AT, and EM performed the experiments. TB and RB contributed to the design of the study. CA and IH initiated many of the experiments and performed the analysis. CA wrote the manuscript together with TK.

### Conflict of Interest Statement

The authors declare that the research was conducted in the absence of any commercial or financial relationships that could be construed as a potential conflict of interest.

## Supplementary Material

Supplement Figure 1 FACS sorting strategy of bone marrow and neutrophils from blood. Bone marrow and mature neutrophils from blood were sorted based on CD11b and CD16 expression under cold conditions and with a small nozzle. **(A)** Representative flow cytometry images of gating strategy for FACS sorting. **(B)** Representative images of progenitors cells isolated from the bone marrow after FACS sorting stained by May-Giemsa.

Supplement Figure 2 Read-out of T cell proliferation in absence or presence of fMLF-activated neutrophils. Purified CFSE-labeled T cells from healthy donors were cultured with anti-CD3 and anti-CD28 antibodies in presence or absence of mature neutrophils from control donors. After 5 days of culture, cells were collected for FACS analysis and stained with anti-CD4 and anti-CD8 antibodies. Lymphocytes were gated based on size (first panel) followed by the exclusion of duplet cells (second panel). The CFSE dilution of CD4^+^ and CD8^+^ was measured and each cell division was gated for the calculation of the precursor frequency to quantify the proliferation.

Supplement Figure 3 Sorted neutrophil progenitors from bone marrow do not suppress CD8^+^ T cell proliferation. Neutrophil progenitors from bone marrow were isolated via FACS sorting based on CD11b and CD16 expression under cold conditions and with a small nozzle. Purified CFSE-labeled T cells from healthy donors (*n* = 6) were cultured with anti-CD3 and anti-CD28 antibodies (white bars), and in presence of mature neutrophils from control donors (black bars, *n* = 6) or sorted neutrophil progenitors from bone marrow (gray bars, *n* = 3) and/or indicated stimuli. Cells were harvested after 5–6 days and analyzed by flow cytometry for CFSE dilution among CD8^+^ T cells. Error bars indicate SEM; ^****^*p* < 0.0001.

Supplement Figure 4 Incubation with FACS antibodies under cold conditions does not impair ROS production. Neutrophils were left unlabeled at RT (white bars) or at 4°C (gray bars) or labeled with anti-CD11b and anti-CD16 antibodies at 4°C (black bars) for 30 min. Cells were stimulated with the indicated stimuli and production of H_2_O_2_ was determined by measuring Amplex Red conversion into fluorescent Resorufin (*n* = 3).

Supplement Figure 5 Sorted mature neutrophils do not suppress CD8^+^T cell proliferation. Purified CFSE-labeled T cells from healthy donors were cultured with anti-CD3 and anti-CD28 antibodies (white bars), and in presence of unsorted (black bars) or sorted (gray bars) mature neutrophils from control donors and/or indicated stimuli (*n* = 3). Sort was based on size (FSC/SSC) under RT conditions and a big nozzle. Cells were harvested after 5–6 days and analyzed by flow cytometry for CFSE dilution among CD8^+^ T cells. Error bars indicate SEM; ^**^*p* < 0.01.

Supplement Figure 6 FACS analysis of bone marrow pellet after density centrifugation. The surface marker expression of CD11b and CD16 was measured by flow cytometry analysis of cells in the bone marrow pellet after density centrifugation. Neutrophil progenitors were first gated based on size **(Left)** and then gated based on the expression of CD11b and CD16 **(Right)**. Shown are representative FACS analysis images (*n* = 3).

Supplement Figure 7 Neutrophils progenitors from BM pellet fraction do not suppress CD8^+^T cell proliferation. Purified CFSE-labeled T cells from healthy donors were cultured with anti-CD3 and anti-CD28 antibodies (white bars, *n* = 6), and in presence of mature neutrophils from blood (black bars, *n* = 6) or neutrophil progenitors from the bone marrow pellet (gray bars, *n* = 3) and/or indicated stimuli. Cells were harvested after 5–6 days and analyzed by flow cytometry for CFSE dilution among CD8^+^ T cells. Error bars indicate SEM; ^****^*p* < 0.0001.

Supplement Figure 8 Bone marrow cell fractions obtained by discontinuous Percoll fractionation show cell heterogeneity. **(A)** Schematic drawing of the set-up of the discontinuous Percoll fractionation. Bone marrow was placed upon a two-layer Percoll gradient of densities 1.065 and 1.080 g/mL, generating four fractions after centrifugation. **(B)** Gating strategy of flow cytometry analysis of the four BM cell fractions. Shown are representative FACS analysis images of the granulocyte gating based on size (FSC/SSC). **(C)** The percentage of the different neutrophil progenitors within the cell fractions (indicated by number on the x-axis) were measured by flow cytometry based on CD11b and CD16 expression within the granulocyte gate shown in **(B)**. **(D)** The indicated cell fractions and neutrophils from blood were stimulated with the indicated stimuli and production of H_2_O_2_ was determined by measuring Amplex Red conversion into fluorescent Resorufin (*n* = 2–4).

Supplement Figure 9 FACS analysis of mature neutrophils and neutrophil progenitors before and after CD16+ MACS isolation. The surface marker expression of CD11b and CD16 was measured by flow cytometry analysis of both mature neutrophils from blood **(Left)** and neutrophil progenitors from BM pellet **(Right)** before and after CD16 positive MACS isolation. Shown are representative FACS analysis images (*n* = 3).

Supplement Figure 10 Only the CD16^+^ neutrophil progenitors can suppress CD8^+^T cell proliferation. CD16 positive cells were isolated via MACS isolation from mature neutrophils from blood and neutrophil progenitors from BM pellet. Purified CFSE-labeled T cells from healthy donors (*n* = 4) were cultured with anti-CD3 and anti-CD28 antibodies (white bars), and in presence of mature neutrophils from control donors (black bars, *n* = 6), CD16^+^ mature neutrophils (gray bars, *n* = 6), total BM pellet fraction (dark green bars, *n* = 4), CD16^+^ (green bars, *n* = 4) or CD16^−^ (light green bars, *n* = 4) progenitors from BM pellet and/or indicated stimuli. Cells were harvested after 5–6 days and analyzed by flow cytometry for CFSE dilution among CD8^+^ T cells. ^*^*p* < 0.05, ^**^*p* < 0.0001.
