# Supplementary figures and images for: Neutrophils as Suppressors of T Cell Proliferation: Does Age Matter?
Source: Front Immunol. 2019 Sep 11;10:2144. doi: 10.3389/fimmu.2019.02144 (PMC6749034; doi:10.3389/fimmu.2019.02144)

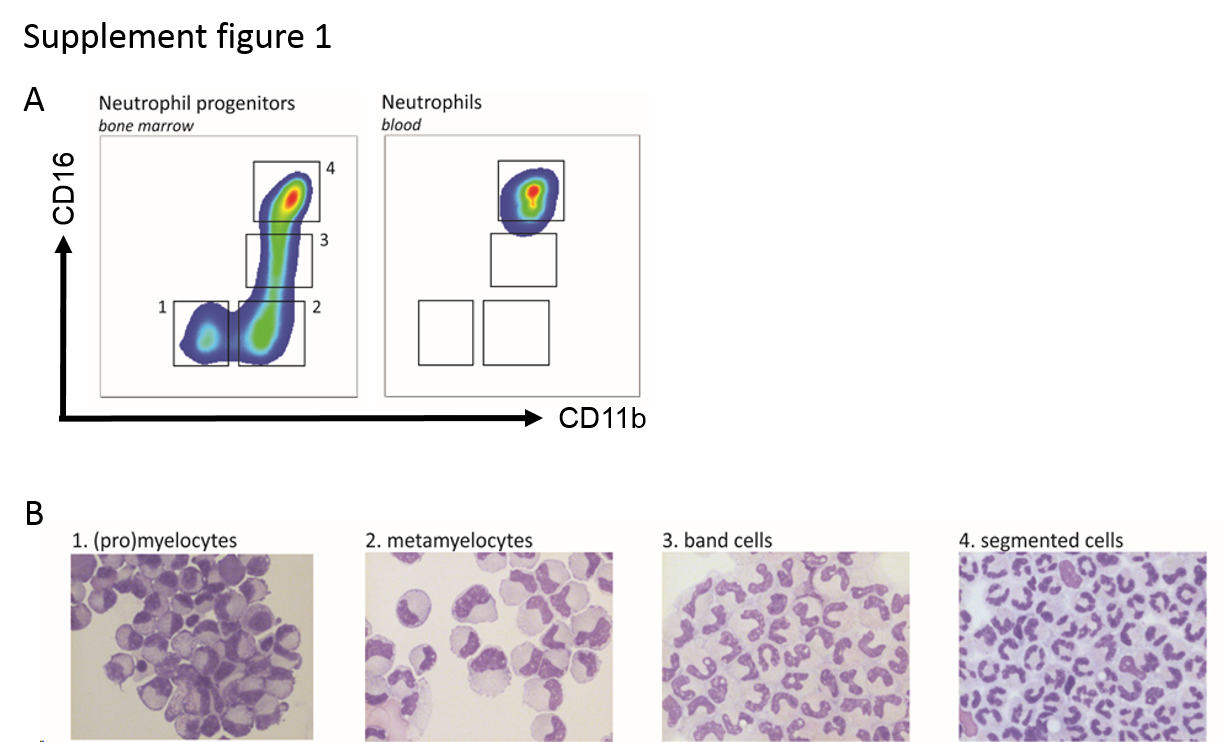

Supplement: Supplement Figure 1 — FACS sorting strategy of bone marrow and neutrophils from blood. Bone marrow and mature neutrophils from blood were sorted based on CD11b and CD16 expression under cold conditions and with a small nozzle. (A) Representative flow cytometry images of gating strategy for FACS sorting. (B) Representative images of progenitors cells isolated from the bone marrow after FACS sorting stained by May-Giemsa. [file Image_1.TIF]

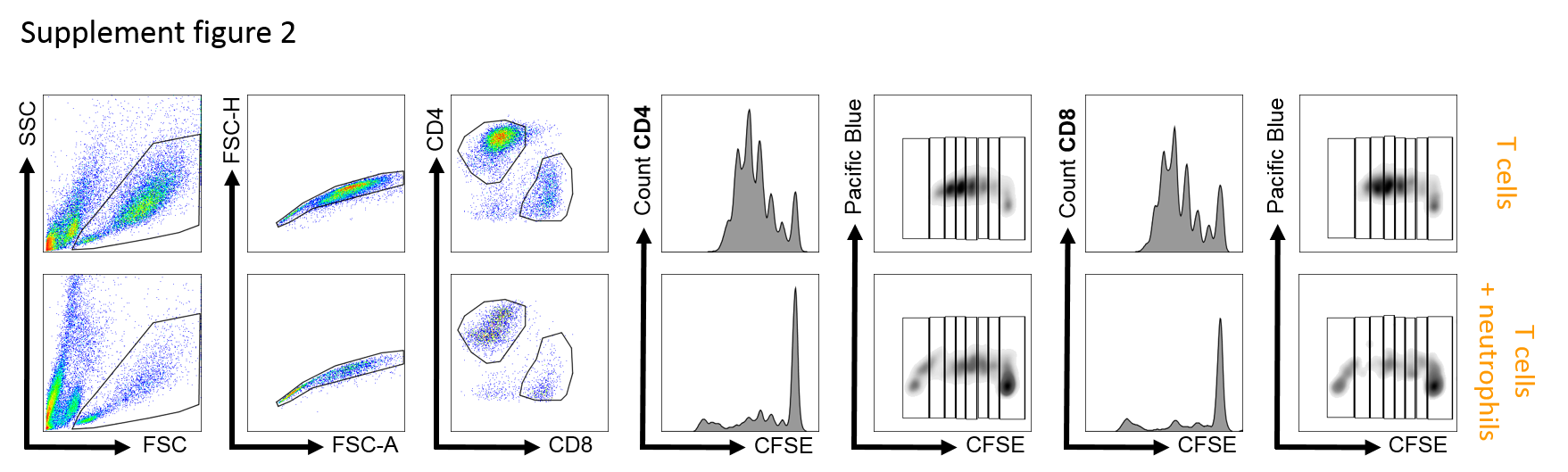

Supplement: Supplement Figure 2 — Read-out of T cell proliferation in absence or presence of fMLF-activated neutrophils. Purified CFSE-labeled T cells from healthy donors were cultured with anti-CD3 and anti-CD28 antibodies in presence or absence of mature neutrophils from control donors. After 5 days of culture, cells were collected for FACS analysis and stained with anti-CD4 and anti-CD8 antibodies. Lymphocytes were gated based on size (first panel) followed by the exclusion of duplet cells (second panel). The CFSE dilution of CD4+ and CD8+ was measured and each cell division was gated for the calculation of the precursor frequency to quantify the proliferation. [file Image_2.TIF]

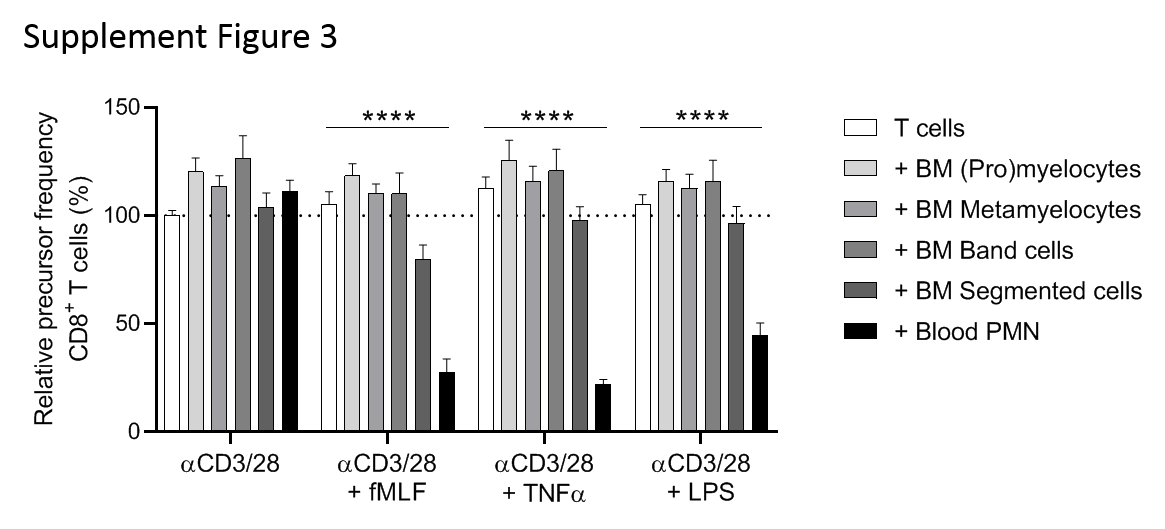

Supplement: Supplement Figure 3 — Sorted neutrophil progenitors from bone marrow do not suppress CD8+ T cell proliferation. Neutrophil progenitors from bone marrow were isolated via FACS sorting based on CD11b and CD16 expression under cold conditions and with a small nozzle. Purified CFSE-labeled T cells from healthy donors (n = 6) were cultured with anti-CD3 and anti-CD28 antibodies (white bars), and in presence of mature neutrophils from control donors (black bars, n = 6) or sorted neutrophil progenitors from bone marrow (gray bars, n = 3) and/or indicated stimuli. Cells were harvested after 5–6 days and analyzed by flow cytometry for CFSE dilution among CD8+ T cells. Error bars indicate SEM; ****p < 0.0001. [file Image_3.TIF]

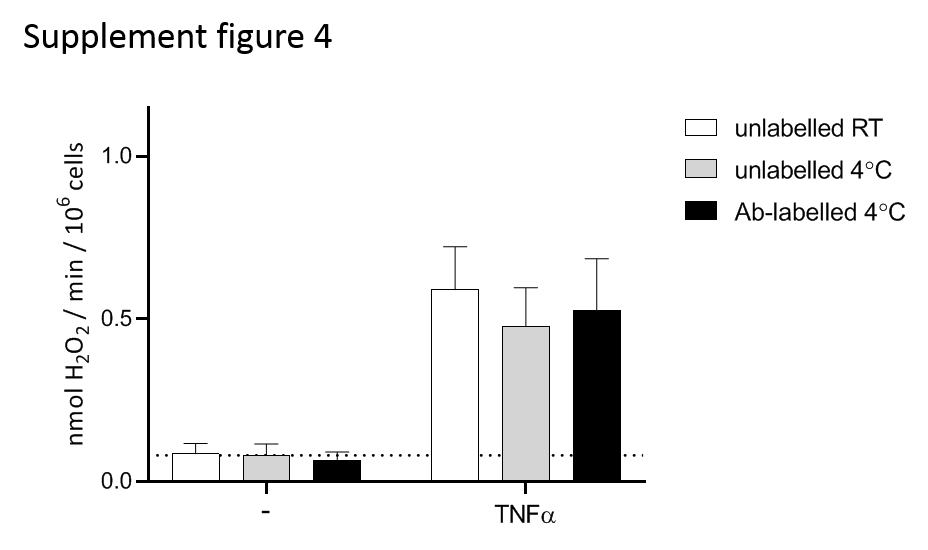

Supplement: Supplement Figure 4 — Incubation with FACS antibodies under cold conditions does not impair ROS production. Neutrophils were left unlabeled at RT (white bars) or at 4°C (gray bars) or labeled with anti-CD11b and anti-CD16 antibodies at 4°C (black bars) for 30 min. Cells were stimulated with the indicated stimuli and production of H2O2 was determined by measuring Amplex Red conversion into fluorescent Resorufin (n = 3). [file Image_4.TIF]

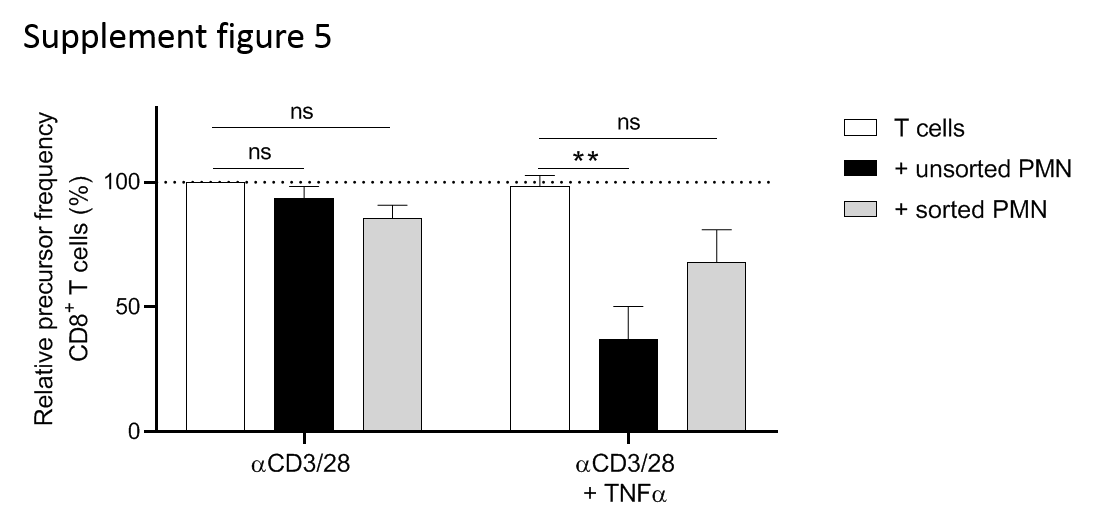

Supplement: Supplement Figure 5 — Sorted mature neutrophils do not suppress CD8+T cell proliferation. Purified CFSE-labeled T cells from healthy donors were cultured with anti-CD3 and anti-CD28 antibodies (white bars), and in presence of unsorted (black bars) or sorted (gray bars) mature neutrophils from control donors and/or indicated stimuli (n = 3). Sort was based on size (FSC/SSC) under RT conditions and a big nozzle. Cells were harvested after 5–6 days and analyzed by flow cytometry for CFSE dilution among CD8+ T cells. Error bars indicate SEM; **p < 0.01. [file Image_5.TIF]

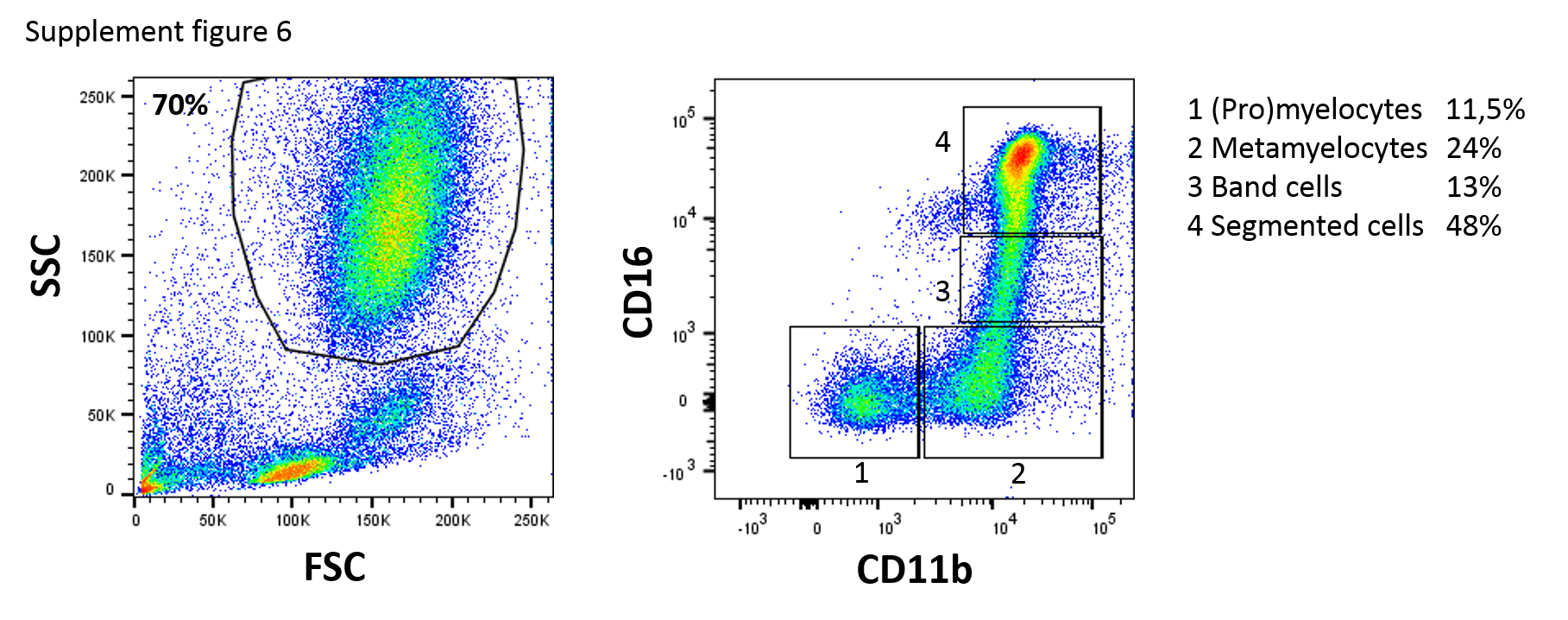

Supplement: Supplement Figure 6 — FACS analysis of bone marrow pellet after density centrifugation. The surface marker expression of CD11b and CD16 was measured by flow cytometry analysis of cells in the bone marrow pellet after density centrifugation. Neutrophil progenitors were first gated based on size (Left) and then gated based on the expression of CD11b and CD16 (Right). Shown are representative FACS analysis images (n = 3). [file Image_6.TIF]

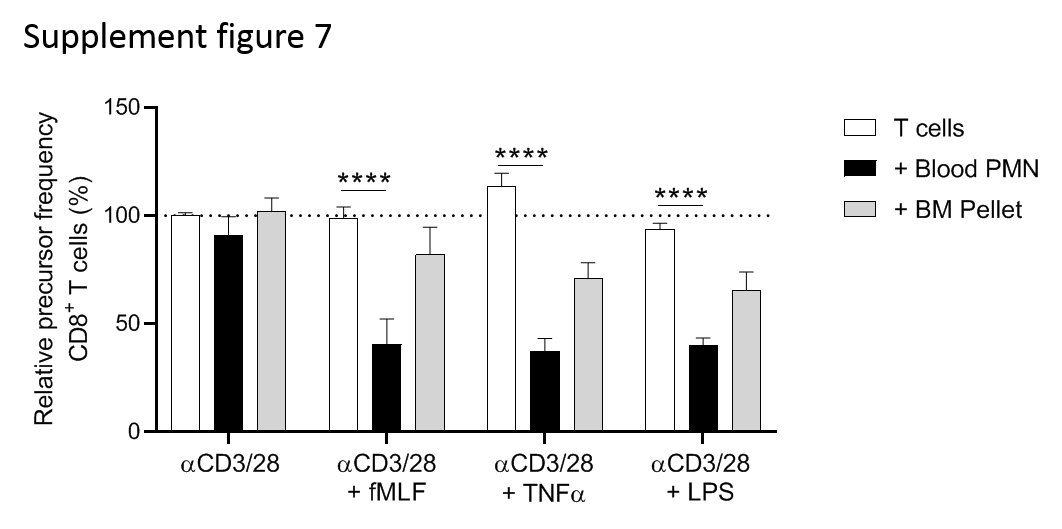

Supplement: Supplement Figure 7 — Neutrophils progenitors from BM pellet fraction do not suppress CD8+T cell proliferation. Purified CFSE-labeled T cells from healthy donors were cultured with anti-CD3 and anti-CD28 antibodies (white bars, n = 6), and in presence of mature neutrophils from blood (black bars, n = 6) or neutrophil progenitors from the bone marrow pellet (gray bars, n = 3) and/or indicated stimuli. Cells were harvested after 5–6 days and analyzed by flow cytometry for CFSE dilution among CD8+ T cells. Error bars indicate SEM; ****p < 0.0001. [file Image_7.TIF]

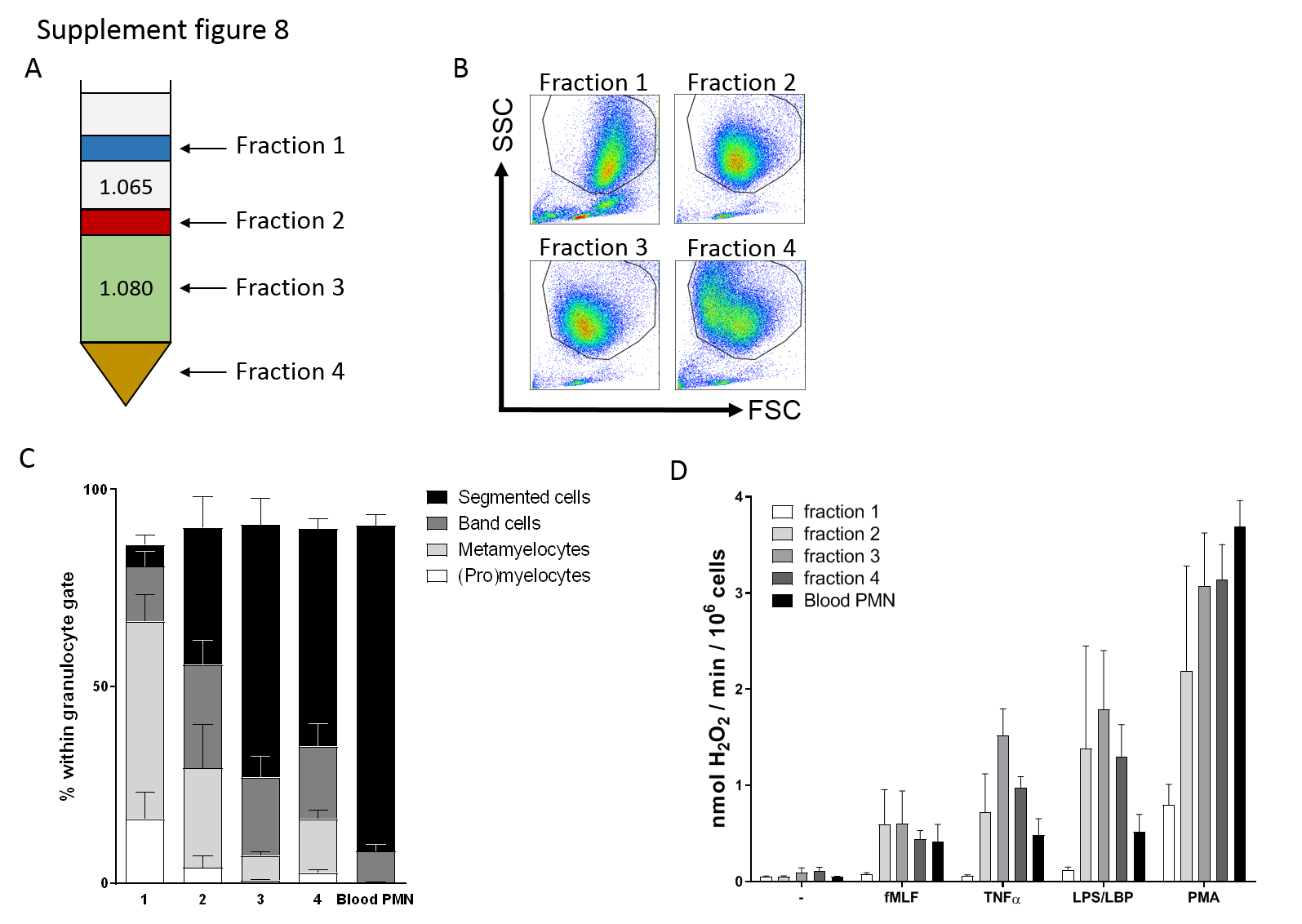

Supplement: Supplement Figure 8 — Bone marrow cell fractions obtained by discontinuous Percoll fractionation show cell heterogeneity. (A) Schematic drawing of the set-up of the discontinuous Percoll fractionation. Bone marrow was placed upon a two-layer Percoll gradient of densities 1.065 and 1.080 g/mL, generating four fractions after centrifugation. (B) Gating strategy of flow cytometry analysis of the four BM cell fractions. Shown are representative FACS analysis images of the granulocyte gating based on size (FSC/SSC). (C) The percentage of the different neutrophil progenitors within the cell fractions (indicated by number on the x-axis) were measured by flow cytometry based on CD11b and CD16 expression within the granulocyte gate shown in (B). (D) The indicated cell fractions and neutrophils from blood were stimulated with the indicated stimuli and production of H2O2 was determined by measuring Amplex Red conversion into fluorescent Resorufin (n = 2–4). [file Image_8.TIF]

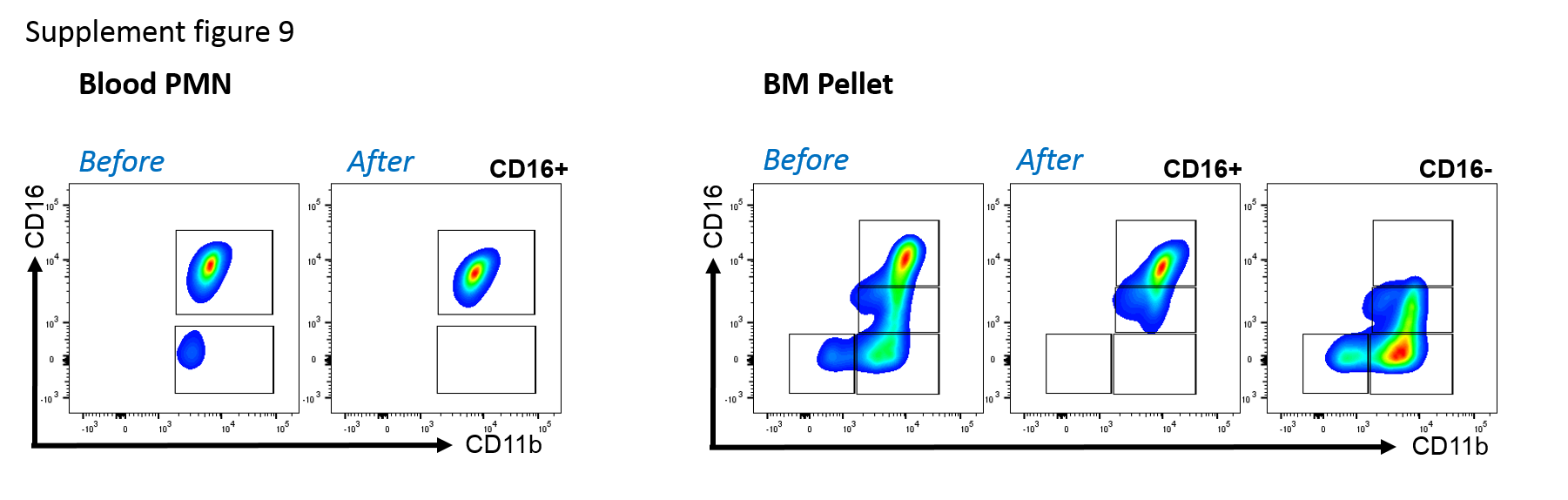

Supplement: Supplement Figure 9 — FACS analysis of mature neutrophils and neutrophil progenitors before and after CD16+ MACS isolation. The surface marker expression of CD11b and CD16 was measured by flow cytometry analysis of both mature neutrophils from blood (Left) and neutrophil progenitors from BM pellet (Right) before and after CD16 positive MACS isolation. Shown are representative FACS analysis images (n = 3). [file Image_9.TIF]

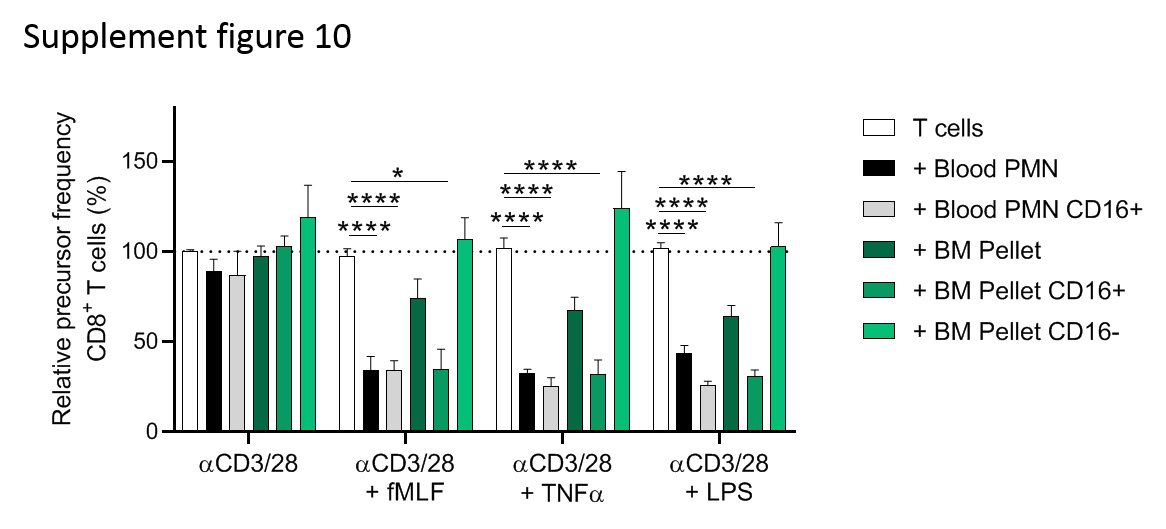

Supplement: Supplement Figure 10 — Only the CD16+ neutrophil progenitors can suppress CD8+T cell proliferation. CD16 positive cells were isolated via MACS isolation from mature neutrophils from blood and neutrophil progenitors from BM pellet. Purified CFSE-labeled T cells from healthy donors (n = 4) were cultured with anti-CD3 and anti-CD28 antibodies (white bars), and in presence of mature neutrophils from control donors (black bars, n = 6), CD16+ mature neutrophils (gray bars, n = 6), total BM pellet fraction (dark green bars, n = 4), CD16+ (green bars, n = 4) or CD16− (light green bars, n = 4) progenitors from BM pellet and/or indicated stimuli. Cells were harvested after 5–6 days and analyzed by flow cytometry for CFSE dilution among CD8+ T cells. *p < 0.05, **p < 0.0001. [file Image_10.TIF]
